# Poly(ADP-ribose) polymerase 1 regulates mitochondrial DNA repair in an NAD-dependent manner

**DOI:** 10.1016/j.jbc.2021.100309

**Published:** 2021-01-19

**Authors:** Geoffrey K. Herrmann, William K. Russell, Nisha J. Garg, Y. Whitney Yin

**Affiliations:** 1 Department of Biochemistry and Molecular Biology, University of Texas Medical Branch, Galveston, Texas, USA; 2 Sealy Center for Structural Biology, University of Texas Medical Branch, Galveston, Texas, USA; 3 Department of Microbiology and Immunology, University of Texas Medical Branch, Galveston, Texas, USA; 4 Department of Pharmacology and Toxicology, University of Texas Medical Branch, Galveston, Texas, USA

**Keywords:** ADP-ribosylation, DNA repair, DNA polymerase, protein–protein interaction, protein–DNA interaction, western blot, DNA synthesis, post-translational modification, ACN, acetonitrile, BER, base excision repair, BME, β-mercaptoethanol, EMSA, electrophoretic mobility shift assay, FA, formic acid, hPARG, human poly(ADP-Ribose) glycohydrolase, mtDNA, mitochondrial DNA, PARP1, Poly(ADP-Ribose) polymerase 1, Pol γ, mitochondrial DNA polymerase, TBST, Tris-buffered saline supplemented with Tween-20, WB, Western blot

## Abstract

Mitochondrial DNA is located in organelle that house essential metabolic reactions and contains high reactive oxygen species. Therefore, mitochondrial DNA suffers more oxidative damage than its nuclear counterpart. Formation of a repair enzyme complex is beneficial to DNA repair. Recent studies have shown that mitochondrial DNA polymerase (Pol γ) and poly(ADP-ribose) polymerase 1 (PARP1) were found in the same complex along with other mitochondrial DNA repair enzymes, and mitochondrial PARP1 level is correlated with mtDNA integrity. However, the molecular basis for the functional connection between Pol γ and PARP1 has not yet been elucidated because cellular functions of PARP1 in DNA repair are intertwined with metabolism *via* NAD+ (nicotinamide adenosine dinucleotide), the substrate of PARP1, and a metabolic cofactor. To dissect the direct effect of PARP1 on mtDNA from the secondary perturbation of metabolism, we report here biochemical studies that recapitulated Pol γ PARylation observed in cells and showed that PARP1 regulates Pol γ activity during DNA repair in a metabolic cofactor NAD^+^ (nicotinamide adenosine dinucleotide)-dependent manner. In the absence of NAD^+^, PARP1 completely inhibits Pol γ, while increasing NAD^+^ levels to a physiological concentration that enables Pol γ to resume maximum repair activity. Because cellular NAD+ levels are linked to metabolism and to ATP production *via* oxidative phosphorylation, our results suggest that mtDNA damage repair is coupled to cellular metabolic state and the integrity of the respiratory chain.

The oxidative environment of mitochondria results in more damage on mitochondrial DNA (mtDNA) than its nuclear counterpart (1, 2). Most of the oxidative damage to mtDNA is restored *via* base excision repair (mtBER), which, like nuclear BER, can be functionally grouped into three distinct steps: (1) lesion recognition, (2) gap tailoring, and (3) DNA synthesis/ligation (3). Although mtBER lesion recognition occurs similarly to nuclear BER, the pathway subsequently diverges. For example, a mitochondria-specific endo/exonuclease G, excises a dinucleotide from the 5’-end of the DNA intermediate in gap-tailoring, generating a DNA gap larger than a single nucleotide. In contrast to specialized DNA polymerases in the nucleus, the mitochondrial DNA polymerase (Pol γ) that is responsible for DNA replication also acts in gap-filling DNA synthesis in mtBER (4). The regulatory mechanisms that govern Pol γ functional conversion have not been illustrated.

Poly(ADP-Ribose) (PAR) polymerase 1 (PARP1) is a first responder to DNA strand breaks by serving as a key scaffolding protein in recruitment of other repair enzymes to the lesion site and assembling a repair complex around the lesion DNA (5, 6, 7, 8). In the complex, PARP1 uses NAD^+^ to catalyze poly(ADP-ribosylation) on itself (auto-PARylation) and target proteins (*trans*-PARylation). PARylation activity of PARP1 was discovered in nucleus more than 50 years ago, then later found in mitochondria, first in extracts of Xenopous oocytes (9). Since then, significant PARP1 levels have been demonstrated in mitochondria of rat liver (10), murine cardiomyocytes (11, 12), and human Sertoli and alveolar basal epithelial cells (12, 13), and PARP1 localization and activity in association with mtDNA in mitochondrial matrix was documented by various approaches (12, 14, 15). Additionally, PARP-1 was found to be co-localized with Pol γ in cardiomyocytes and alveolar basal epithelial cells (A549), and Pol γ was PARylated in these cells (11, 13). Many other ADP-ribosylated mitochondrial proteins have also been discovered (10, 15, 16, 17). An interactor molecule, Mitofilin, has been shown to control PARP-1 transport into mitochondria (18), supporting the notion that these proteins are modified inside mitochondria rather than being transported as PARylated species. These studies provide substantial evidence for mitochondrial localization of PARP-1 and imply that PARP-1 may affect mtDNA *via* Pol γ.

Despite its mitochondrial localization, the function of PARP-1 during mtDNA repair is disputed in cellular studies. For instance, mtDNA integrity increased when PARP-1 was knocked down (13) and mtDNA content declined during PARP-1 overexpression (11), suggesting PARP-1 negatively impacts mtDNA integrity. Opposing results show that mtDNA stability reduces following PARP1 knockdown (18), PARP-1 deletion increased DNA damage under oxidative conditions (19), and the addition of PARP-1 inhibitor augments UV-induced damage on mtDNA (20), all suggesting PARP1 contributes positively to mtDNA integrity. The complicated PARP-1 cellular effects are likely because of multiple roles of NAD^+^ as substrate of PARP-1 and a metabolic cofactor. Intramitochondrial PARP1 resulted in NAD^+^ depletion and lead to metabolic disorder, reduced ATP production, and cell death (15).

To begin dissecting PARP1 function in mtDNA repair, away from its role in metabolism, we conducted biochemical studies to investigate the activity of PARP1 during mtDNA repair and how it affects Pol γ functionality. We report here that PARP1 differentially affects Pol γ in replication and repair. While having little impact on Pol γ during replication, PARP1 regulates Pol γ repair activity in a NAD-dependent manner. Pol γ is a substrate for PARP1, and PARylation is essential for PARP1 regulation of Pol γ repair activities. Our studies thus revealed a crosstalk between mtDNA repair and cellular metabolism.

## Results

### PARP1 interacts with Pol γ on DNA repair intermediates

To recapitulate the interaction of PARP1 and Pol γ in mitochondrial lysate by co-immunoprecipitation (11), we performed binding assays utilizing purified PARP1 and Pol γ with or without a 75-nt dumbbell DNA containing a 3-nt gap. The gapped DNA was designed based on activity of the mitochondrial-specific endo/exonuclease G, which excises two-nucleotides from the 5’-end of the gap of duplex DNA in mtBER, thereby creating 3-nt gapped DNA repair intermediate (21, 22, 23) that mimics a mtDNA BER intermediate (Fig. S1*A*). The 25 bp upstream of the gap meets the minimum required length for binding of Pol γ, and the 6 bp downstream of DNA gap was found to be sufficient for binding of PARP1 (24, 25). Pol γ, a heterotrimeric holoenzyme consisting of a catalytic subunit Pol γA and a dimeric accessory subunit Pol γB, has high affinity to the 3’-end of the primer DNA.

Binary complexes of Pol γ-DNA and PARP1-DNA were first measured using nano-isothermocalorimetry (nano-ITC). At 25 °C, the enthalpy for PARP1-DNA binary complex was −58.4 kcal/mol, and the dissociation constant (K_d_) was calculated to be 36.4 nM (Fig. S1*A*). There was no measurable ΔH for Pol γ binding to DNA at 25 °C (Fig. S1*B*). Because Pol γ was shown to bind this DNA substrate under these conditions (discussed in the following paragraph), we concluded that the binding is isothermal at 25 °C, which simplified evaluation of the Pol γ–PARP1–DNA ternary complex. The calculated K_d_ for the ternary complex was 111 nM (Fig. S1*C*) with enthalpy ΔH_PARP1:Pol γ:DNA_ −61.4 kcal/mol, similar to the measured enthalpy of the PARP1–DNA binary complex. These results suggest formation of PARP1–Pol γ–DNA ternary complex, and binding is entropically driven (Fig. S1*D*). The lower affinity of the ternary complex relative to either binary complex (24) may be because of steric hindrance of Pol γA *Pol* and PARP1 Zn2 domain simultaneously binding to the same DNA gap (26, 27, 28). Protein–protein interaction was measured by titrating PARP-1 into Pol γ (5 μM). No enthalpy was detected at maximum concentration of PARP-1 (final 10 μM), indicating the two proteins have low or no affinity to each other, with a Kd value greater than 10 μM (Fig. S1*E*). In comparision to their Kd value of 111 nM in the presence of DNA, the binding studies suggest the Pol γ–PARP1 interaction is mediated by DNA.

As multicomponent binding constants are difficult to deconvolute from isothermal titration calorimetry (ITC) data, we performed electrophoretic mobility shift assays (EMSAs) to visualize complex formation directly. EMSA supershift assays were performed by titrating increasing amounts of PARP1 into a constant quantity of Pol γ–DNA complex. A super-shifted species emerged that migrated differently from either PARP1–DNA or Pol γ–DNA complexes. The quantity of this species is positively correlated with PARP1 concentration (Fig. S2, *A* and *B*). To identify the components in the super-shifted species, Western blot (WB) was performed using antibody against Pol γ. When PARP1 is added, the super-shifted band contains Pol γ. The binding assays were carried out using substoichiometric amount of Pol γ to DNA (2:3 M ratio) so that only one Pol γ is bound to each DNA substrate. Therefore, the super-shifted Pol γ-DNA complex most likely also contains PARP1. The super-shifted species dissociated upon addition of NAD back to Pol γ-DNA binary complex (Fig. S2*C*, lane 4–5), and addition of PARP1 inhibitor, Olaparib, prevented NAD^+^-induced dissociation (Fig. S2*C*, lane 10). These results suggest that PARP1 protein forms a stable complex with Pol γ–DNA, and this binding is decreased by PARylation activity. Results from ITC and EMSA results indicating that Pol γ and PARP1 form a complex on the gapped DNA are in good agreement with *in vivo* observations of PARP1 co-localization with Pol γ in mitochondria by co-immunoprecipitation (11) and proximity ligation assays (13).

### Pol γ *trans*-PARylation occurs on DNA repair intermediates

To examine whether co-localization with PARP1 results in PARylation of Pol γ, WB analyses were carried out using antibodies against Pol γ, PARP-1, and PAR (poly(ADP-ribose)). A DNA with accessible 5’end is essential for PARP1 robust PARylation activity; the minimum length of DNA for PARP1 activity is 8 bp (29). To determine Pol γ PARylation and whether co-localization with PARP1 is essential for the activity, we used two DNA substrates, the dumbbell 3-nt gapped DNA that can accommodate both PARP1 and Pol γ shown by ITC and EMSA analyses and an 8 bp self-annealing DNA duplex that is sufficient to stimulate PARP1 activity but is only long enough to bind PARP1 alone (29).

**Figure 1:**
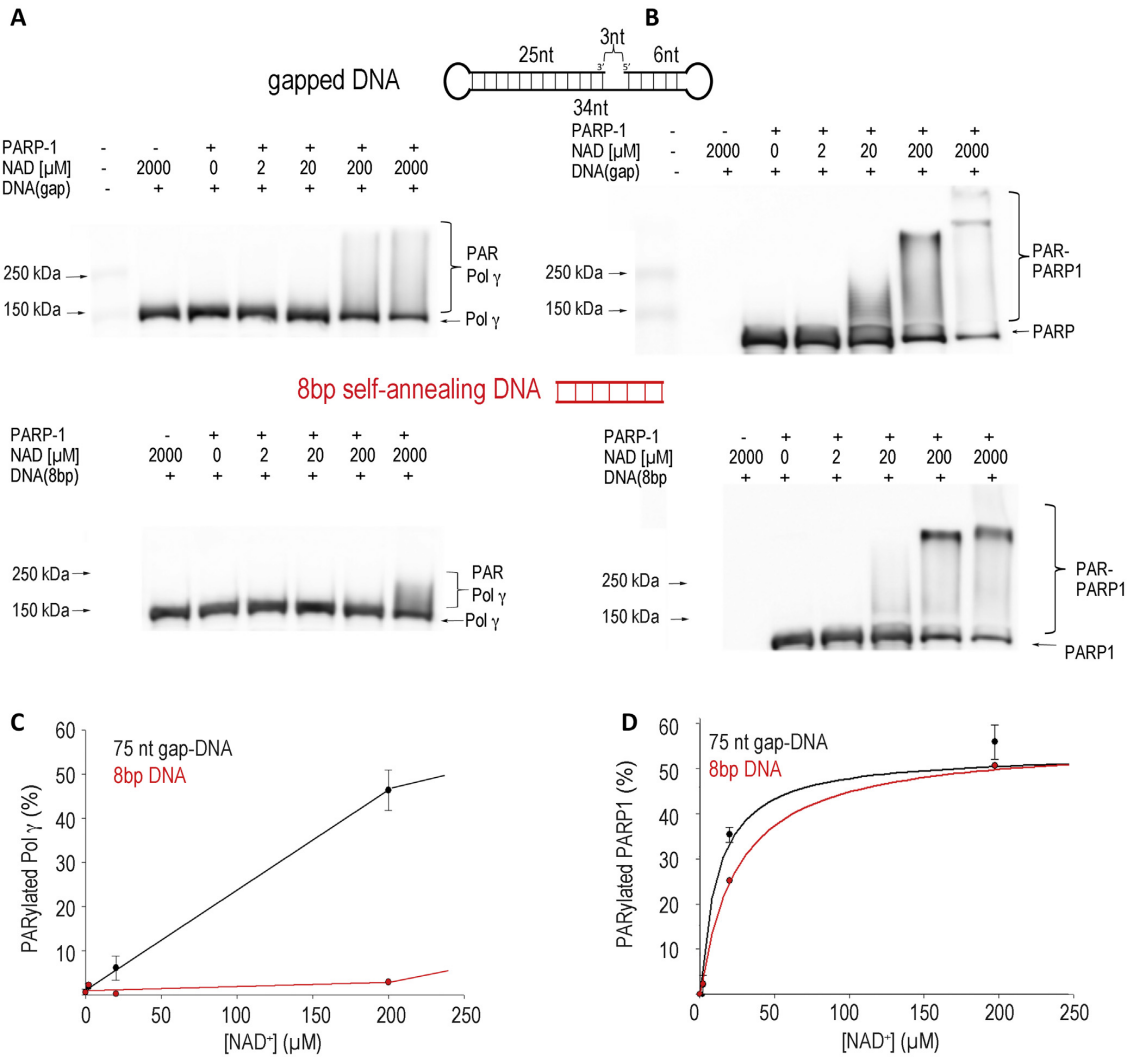
**DNA-dependent Pol γ *trans*-PARylation.***Trans*-PARylation assays were performed by incubating Pol γ (1 μM) the indicated DNA substrate (0.75 μM) and NAD concentration prior to addition of PARP1 (1 μM). Western blot analysis using antibodies against PolgA was performed (*A*) and quantified (*C*) to evaluate *trans*-PARylation. The membrane was stripped, re-probed with antibodies against PARP1 (*B*) and quantified (*D*) to evaluate auto-PARylation. PARP1, Poly(ADP-Ribose) polymerase 1; Pol γ, mitochondrial DNA polymerase.

PARylation reaction products could potentially contain both PARylated and unmodified PARP1 and Pol γ. The heterogeneous nature of the PAR chains results in smearing on denaturing PAGE, thus PARylated Pol γ and PARP1 species could potentially overlap on the gel. Therefore, WBs were performed using antibodies against PARP1 and Pol γ sequentially on the same membrane to distinguish each PARylated species. PARylation reactions were carried out at increasing concentrations of NAD^+^ (20–2000 μM). The WB showed that PARP1 auto-PARylation was equally robust on both DNA substrates and was clearly evident at 20 μM NAD^+^ (Fig. 1*B*, most clearly evidenced by the loss of the unmodified species in the discrete band). Auto-PARylation intensified with increasing concentrations of NAD^+^, plateauing around a physiological concentration of NAD^+^ at 200 μM (Fig. 1, *B* and *D*) (30, 31). By contrast, *trans*-PARylation of Pol γ differed significantly on the two DNA substrates. On the 8 bp duplex, only a small amount of short PAR chains was observed and only at 2000 μM NAD^+^ (Fig. 1*A*), whereas on the gapped DNA, Pol γ PARylation began at 20 μM NAD^+^ and increased sharply at 200 μM NAD^+^ (Fig. 1*C*). No PARylation was observed in the absence of DNA (Fig. S6, furthest lane on the right). This follows logically as PARP1 catalytic activity is stimulated >1000 fold in the presence of DNA (32). These results suggest that at physiological concentrations of NAD^+^, Pol γ was PARylated more readily when bound to the same gapped DNA as PARP1 than when free in solution. Interestingly, lack of *trans*-PARylation did not lead to an increase in auto-PARylated PARP1, suggesting that PARP1 catalyzes auto-PARylation and *trans*-PARylation as two separate reactions and without preference.

The results indicate that Pol γ is a *bona fide* substrate for PARP1, and *trans*-PARylation of Pol γ shown in cellular studies (11, 13) can be recapitulated *in vitro* without involvement of other proteins.

### PARP1 modulates Pol γ gap-filling activity in a NAD^+^-dependent manner

**Figure 2:**
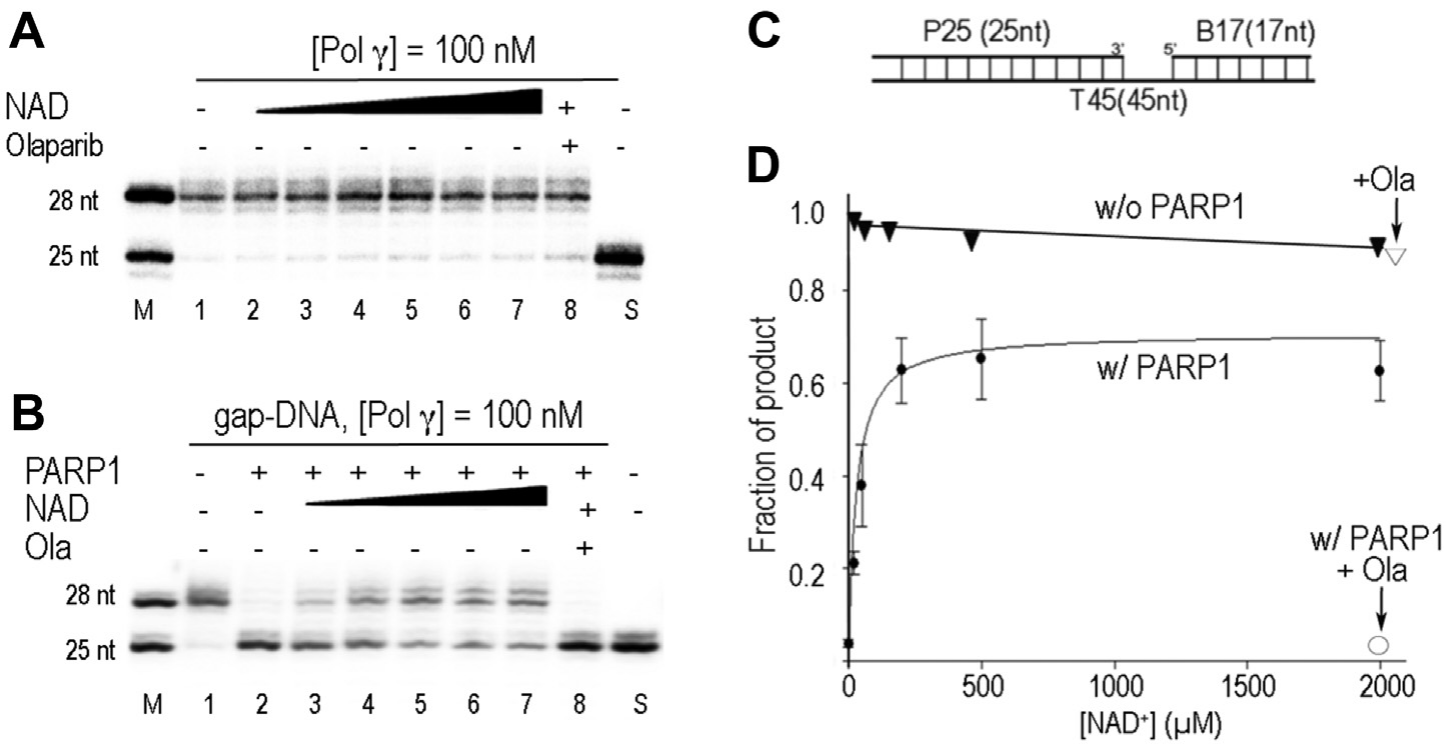
**PARP1 regulates Pol γ gap-filling synthesis.***A*, Pol γ (100 nM) gap-filling synthesis on 3-nt gapped ^32^P-DNA substrate (100 nM) in the absence (Lane 1) or presence of NAD^+^ (20 μM, 50 μM, 200 μM, 500 μM, and 2000 μM, Lanes 2–7) and in the presence of Olaparib (10 μM, Lane 8). Reaction products were separated on 20 or 23% polyacrylamide gels containing 7 M urea and visualized by autoradiography. Lanes M and S denote marker and substrate only lane, respectively. *B*, same as in *A*, except 200 nM PARP1 is present. *C*, substrate for assays containing a 3-nt gap. *D*, quantification of *A* and *B*. PARP1, Poly(ADP-Ribose) polymerase 1; Pol γ, mitochondrial DNA polymerase.

To examine the biological effects of PARP1 interaction and PARylation on Pol γ activity, we investigated Pol γ gap-filling synthesis, an essential activity in DNA repair (Fig. 2*C*) (21, 22). Two products can be seen: the gap-filled product and a product from short strand displacement synthesis that has been previously identified (23). As the strand displacement activity was not a focus of this study, we did not differentiate between the two product bands. Pol γ alone efficiently synthesized into the 3-nt DNA gap (Fig. 2*A*). Addition of stochiometric amount of PARP1 (without NAD^+^) completely inhibited Pol γ gap-filling activity (Fig. 2*B*, Lane 2). The diminished Pol γ activity is most likely because of PARP1 obstructing Pol γ from accessing the template in the DNA gap. To examine the effects of PARylation, NAD^+^ was titrated into the reaction mixture. As NAD^+^ concentration increases, beginning at 50 μM NAD^+^, Pol γ gap-filling activity was gradually recovered (Fig. 2*B*, Lanes 3–7, Fig. 2*D*) and reached a maximum at 200 μM. Further increasing NAD^+^ to 2000 μM offers negligible benefit (Fig. 2, *B* and *D*). NAD^+^ itself has no effect on Pol γ activity (Fig. 2, *A* and *D*). These results, along with those presented in Figure 1, suggest that PARP1 binding inhibits Pol γ activity, and NAD^+^-dependent auto- and *trans*-PARylation under physiological conditions regulates the PARP1–Pol γ interaction in favor of Pol γ activity.

Recent studies suggest that the human nuclear BER polymerase, Pol β, could be localized in mitochondria (32, 33) and therefore could participate in mtBER. We thus tested the effect of PARP1 on Pol β gap-filling activity. Similar to results from Pol γ, Pol β activity is also inhibited by PARP1, and gap-filling synthesis is restored by the addition of NAD^+^ (Fig. S3, *A* and *B*), consistent with the previous report (34). Like Pol γ, Pol β was PARylated at a NAD^+^ concentration (200 μM) much higher than that required (20 μM NAD^+^) for gap-filling synthesis (Fig. S3, *A* and *C*), indicating PARylation of the polymerases is not essential for the activity. Our results suggest BER gap-filling synthesis is regulated by PARP1 and NAD^+^, regardless of which polymerase performs it.

To confirm PARylation is a key regulator for the PARP1–Pol γ interaction, we added to the reaction a PARP inhibitor, Olaparib, which competes with NAD^+^, thus obliterating PARP1 PARylation activity. Olaparib completely abolishes the NAD^+^ rescuing effect for both DNA polymerases tested (Fig. 2*B*, Lane 8; Fig. S3*A*, lane 8), confirming the catalytic activity of PARP1 is involved in regulation of Pol γ and Pol β activities.

The above results imply that during mtDNA BER, PARP1 only enables Pol γ gap-filling synthesis in the presence of apt concentration of NAD^+^, otherwise PARP1 clasps itself on the DNA, acting as a physical barrier preventing mtDNA repair. Our results provide an explanation for the cellular studies that show PARP1 activity inhibitors are detrimental to mtDNA (13) while PARP1 depletion was beneficial in various models of cardiomyopathy (11, 35), and how PARP1 could be beneficial and detrimental to mtBER (11, 13, 18, 19).

### Excessive PARP1 halts Pol γ DNA repair activity

**Figure 3:**
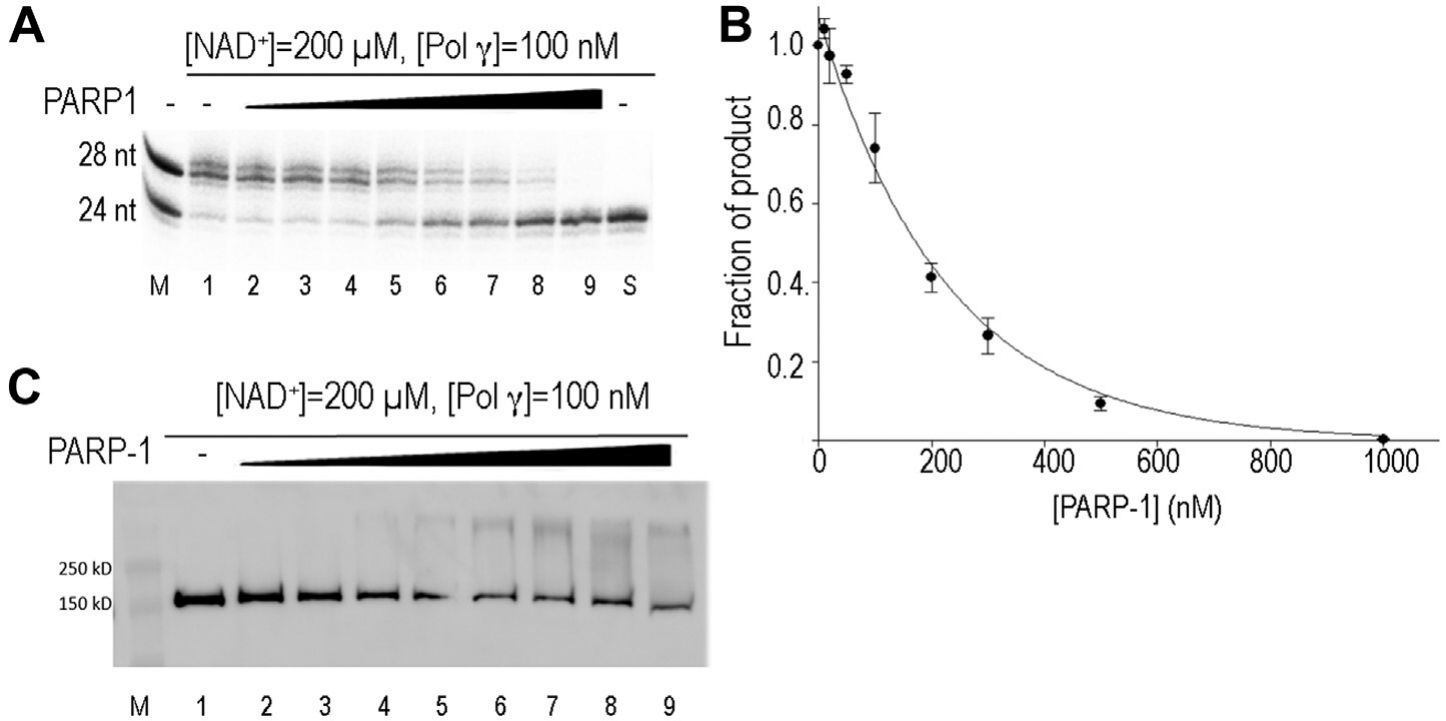
**Effects of excess PARP1 on Pol γ gap-filling activity.***A*, Pol γ (100 nM) gap-filling synthesis on 3-nt gapped 32P-DNA substrate (100 nM) in presence of constant NAD^+^ (200 μM) and increasing concentration of PARP1 (10 nM, 20 nM, 50 nM, 100 nM, 200 nM, 300 nM, 500 nM, 1000 nM, Lanes 2–9). Reaction products were separated on 20 or 23% polyacrylamide gels containing 7 M urea and visualized by autoradiography. *B*, western blot of an identical assay (using nonradiolabeled DNA), probed with antibodies against Pol γ showing Pol γ *trans*-PARylation as a function of PARP1 concentration. *C*, quantification of *A*. PARP1, Poly(ADP-Ribose) polymerase 1; Pol γ, mitochondrial DNA polymerase.

Oxidative stress or infection could induce PARP1 overactivation (11, 15), causing DNA damage. To analyze effects of excessive PARP1 on mtBER, we examined Pol γ gap-filling activity with elevated concentration of PARP1 at a constant NAD^+^ level to mimic PARP1 overexpression under pathological conditions. When assayed at PARP1:Pol γ molar ratios ranging from 1:10 to 10:1, Pol γ gap-filling activity began declining at a 1:1 M ratio and continued exponential decline with increasing PARP1 concentration (Fig. 3, *A* and *C*). Pol γ activity is completely halted at 10-fold molar excess of PARP1 to Pol γ (Fig. 3*A*, lane 9).

The excess molar ratio of PARP1 to Pol γ provides an opportunity to examine the order of auto-PARylation *versus trans*-PARylation, *i.e.*, whether PARP1 preferentially catalyzes auto-PARylation of itself before *trans*-PARylation of substrates with limited NAD^+^. Previous studies show that each PAR chain can contain up to 200 ADP-ribose moieties (36), and each PARP1 molecule has at least 37 PARylation sites (37). Because >60% of our purified PARP1 molecules are active (Fig. 1*B*), if PARP1 preferentially catalyzes auto-PARylation, auto-PARylation could consume all 200 μM NAD^+^ in the reaction when PARP1 concentration exceeds 100 nM, leaving no NAD^+^ for *trans*-PARylation of Pol γ. Under this assumption, we would expect to see PARylated Pol γ diminish when the PARP1 to Pol γ molar ratio is ≥1. On the contrary, Pol γ PARylation does occur at the 1:1 M ratio and was strengthened with increasing concentrations of PARP1 (Fig. 3*B*). Based on this estimation, the result suggests preferential auto-PARylation is less probable.

The above results suggest that PARP1 either PARylates Pol γ before, or simultaneously with, auto-PARylation before dissociation from DNA. In either case, the rate of PARP1 binding to the gapped DNA must be faster than Pol γ gap-filling synthesis to halt its activity. The excess non-PARylated PARP1 correlates well with the decreased activity (Fig. 3, *B* and *C*), supporting the notion that PARylated PARP1 dissociates from the DNA, whereas the non-PARylated species blocks Pol γ activity.

As PAR chains elongate, the negatively charged polymers cause PARylated proteins to dissociate from DNA. Because PARylated PARP1 displays significantly lower catalytic activity (38), we tested activity of PARylated Pol γ. We first use PARP1 to *trans*-PARylate Pol γ, in the presence of 200 μM NAD^+^, then purified PARylated Pol γ from the unmodified polymerase, and used the PARylated species to carry out gap-filling synthesis (Fig. S5, *A* and *B*). Time-dependent products from both modified and unmodified Pol γ were quantified. The synthesis of the full-length products from modified polymerase was at least 2-fold less than that of the unmodified enzyme (Fig. S5*C*), suggesting the modification either reduces catalysis or affinity to DNA. In light of results showing Pol γ displayed normal activity when in the presence of PARP1 and same concentration of NAD simultaneously, the reduced activity of PARylated Pol γ must be because of no or incomplete PARylation. The results, in turn, suggest that gap-filling synthesis occurs at a faster rate than complete *trans*-PARylation of Pol γ.

### LC-MS-MS identification of PARylated amino acids

To pinpoint the location of PARylation and to provide structural understanding of the PARP1–Pol γ interaction, we performed LC-MS-MS following the method established by Zhang *et al.* (37). This method utilizes hydroxylamine to digest the PAR chains down to an -NH group on glutamates and aspartates. The peptides and residues containing an additional 15.0109 Da were identified in MS/MS. Duplicate samples containing Pol γ, PARP1, and the 75 nt gapped DNA, with or without NAD^+^ were analyzed. The threshold for identification of PARylation sites was set at 2-fold above the corresponding sample without NAD^+^.

**Figure 4:**
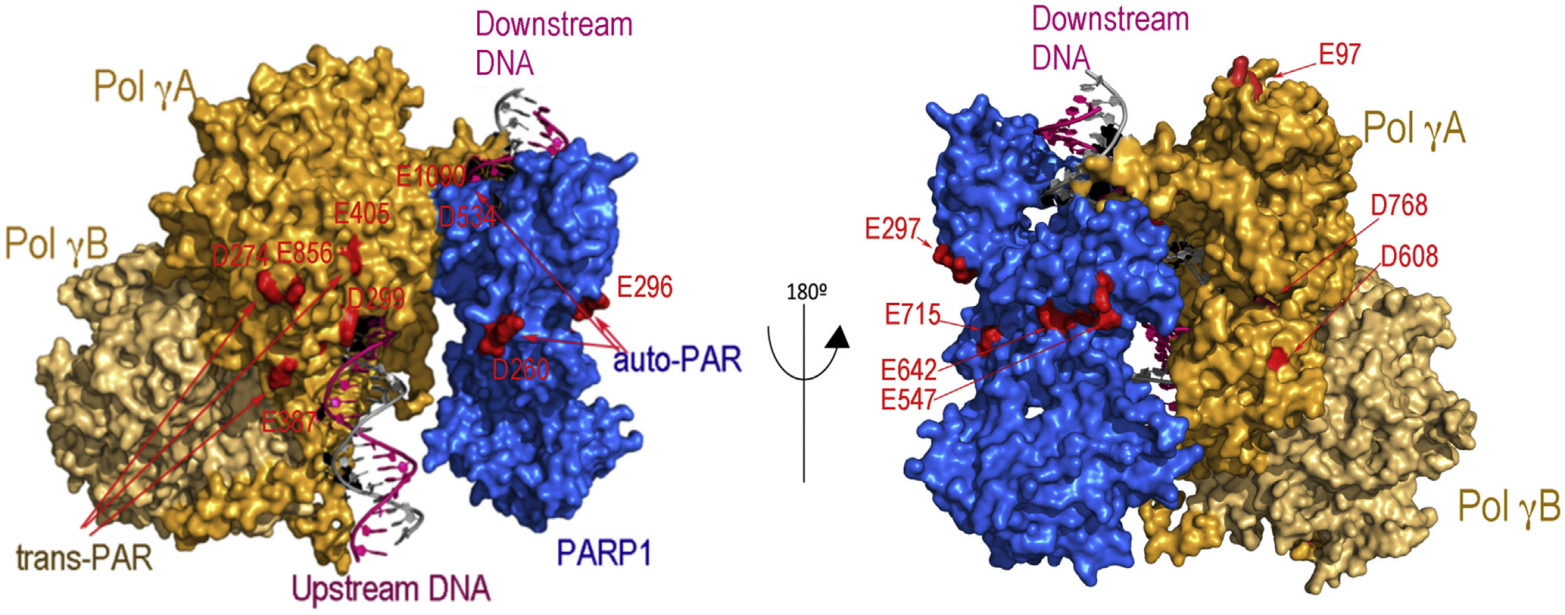
**PARylation sites identified by LC-MS/MS mapped on a model of PARP1 Pol γ-gap DNA complex composited from crystal structures.** PARP1 (*blue*) is located at the 5’-end of the gap and Pol γ at the 3’-end where Pol γA (*orange*) is in close contact with PARP1 and Pol γB (*light orange*) is distal to the DNA gap. The PARylation sites detected are colored *red*. PARP1, Poly(ADP-Ribose) polymerase 1; Pol γ, mitochondrial DNA polymerase.

The peptides from the catalytic subunit Pol γA, accessory subunit Pol γB, and PARP1 were recovered at 87%, 75%, and 82%, respectively. PARylation sites on Pol γA and Pol γB as well as PARP1 were detected. In comparison to the sample without NAD^+^, samples with NAD^+^ displayed increased PARylation sites on Pol γA, Pol γB, and PARP1, which include 12 on PARP1, 16 on Pol γA, and 5 on Pol γB, suggesting the modification sites are correlated with PARylation (Table S3). The detected PARP1 modification sites are a subset of those previously reported from cell lysate (37, 39), perhaps because of the difference in detection thresholds or in purified proteins *versus* more complex cellular environment. Although the method detects PARylation only on glutamate and aspartate residues, the relative PARylation of Pol γ to PARP1 are consistent with that detected by WB (Fig. 1). To visualize the location of the PARylation sites, we composed a model of the Polγ-PARP1-gap DNA complex using crystal structures of Pol γ-DNA (PDB: 4ZTZ) and PARP1-DNA (PDB: 3ODC, 4DQY) by aligning their respective DNA. In the model, the fingers of Pol γA are in close contact with the Zn1 domain PARP1 at the 3’-end and 5’-end of the DNA gap, respectively, and Pol γB is distal to PARP1. When PARylation sites were mapped on the model, most Pol γA PARylation sites are near PARP1. Considering Pol γ does not have a canonical PAR binding motif, the results suggest Pol γ PARylation is enhanced by its close proximity to PARP1 on the gapped DNA (Fig. 4).

As Pol γB is a homodimer, the LC-MS/MS revealed sites cannot be unambiguously identified on each monomer. The number of PARylation sites was then estimated using ^32^P-NAD^+^ in the PARylation reaction followed by enzymatic PAR digestion with human poly(ADP-Ribose) glycohydrolase (hPARG) that leaves the terminal mono(ADP-Ribose) (MAR) on the modified amino acids. The intensity of Pol γA is twice that of Pol γB (Fig. S4). Considering that the total number of amino acids in Pol γA and dimeric Pol γB are comparable, the results indicate that only one monomer of Pol γB, likely the proximal monomer, was modified.

### PARP1 has no effect on Pol γ during DNA replication

**Figure 5:**
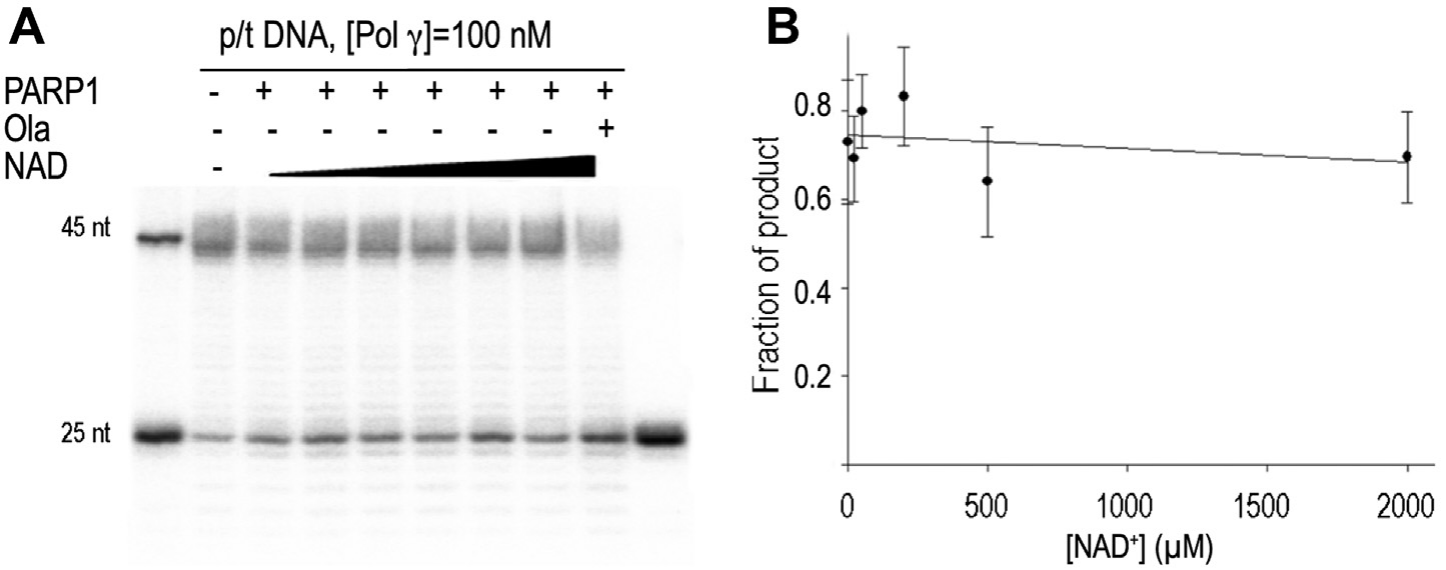
**PARP1 effect on Pol γ replication.***A*, Pol γ (100 nM) synthesis on 25/45 nt primer/template DNA (100 nM) in the absence of NAD^+^ and PARP1 (Lane 1), in the presence of PARP1 (200 nM) but without NAD^+^ (Lane 2), in the presence of PARP1 (200 nM) and increasing concentrations of NAD^+^ (20 μM, 50 μM, 200 μM, 500 μM, and 2000 μM, Lanes 3–7), and in the presence of NAD^+^, PARP1, and Olaparib (10 μM, Lane 8). Reaction products were separated on 20 or 23% polyacrylamide gels containing 7 M urea and visualized by autoradiography. *B*, quantification of *A*. PARP1, Poly(ADP-Ribose) polymerase 1; Pol γ, mitochondrial DNA polymerase.

With the discovery that PARP1 regulates Pol γ DNA repair activity, we next tested the effect of PARP1 on Pol γ DNA replication. The assay was carried out under conditions identical to the gap-filling synthesis experiments except that the DNA substrate is the primer/template, 25/45 nt. In contrast to PARP1’s regulation of Pol γ gap-filling activity, PARP1 displayed little effect on Pol γ during replication (Fig. 5). Increasing NAD^+^ concentration had no impact on Pol γ activity. This result suggests PARP1 does not affect Pol γ during mtDNA replication and that the PARP1–Pol γ interaction is specific to mtDNA repair. This finding is consistent with cellular data which only found PARP1 binding to Pol γ, but not to any of the other mtDNA replication machinery (12).

## Discussion

The closeness of the mitochondrial genome to the electron transfer chain that is known site of electrons release to oxygen and superoxide production causes more oxidative mtDNA damage by reactive oxygen species than chromosomal DNA in nucleus. The levels of mtDNA damage in the heart and brain are negatively correlated with longevity in mammals, but such correlation was not observed for nuclear DNA (40). MtDNA damage repair has therefore profound impact in the ageing process, as well as pathology of cardiac and neurodegenerative diseases (41, 42, 43).

Despite PARP1’s reported conflicting roles on mtDNA repair (11, 19, 20), PARP1 is consistently found in the same complex with Pol γ and other repair enzymes (11, 13). As an initial effort to tease out PARP1 functions in mtDNA repair from its activation induced NAD-depletion and consequential cellular dysfunction, we carried out *in vitro* biochemical studies to investigate PARP1 activity on Pol γ as well as Pol β in mtDNA repair. We found that both polymerases repair activities are strongly regulated by PARP1 in a NAD^+^-dependent manner. PARP1 enables mtDNA repair under physiological concentrations of NAD^+^ while impeding the polymerase’s repair activity at low NAD^+^. Overexpression of PARP1, which occurs under oxidative stress or infection, also inhibits gap-filling activity. This inhibition is likely because of persistent stalling of PARP1 on the broken DNA ends, which thereby hinders DNA repair. Our findings reconcile seemingly opposing effects of PARP1 on mtDNA repair observed in cellular studies (11, 13, 18, 19) and suggest that whether PARP1 is inhibitory to mtDNA BER *in vivo* depends on both the NAD^+^ and PARP1 levels. In the presence of low NAD^+^ or excess PARP1, an inhibitory effect is seen. As sufficient NAD^+^ becomes available (or the excess PARP-1 is removed), this inhibition is reversed, and mtDNA BER proceeds.

### Relationship of metabolism and mtDNA repair

We show here that NAD^+^ is a signaling molecule for PARP1 regulation of Pol γ activity. Besides being a substrate for PARylation, NAD^+^ is also a cofactor for glucose and fatty acids catabolic reactions in which released energy is stored with reduction of NAD^+^ to NADH. Conversion of NADH back to NAD^+^ to ensure continuity of metabolic reactions is accomplished through oxidative phosphorylation, where the protons are removed from NADH, driving the chemical reaction of ATP synthesis. Cellular NAD^+^ level is compartmentalized: mitochondria house the largest NAD^+^ pool at ∼250 μM, higher than that in the nucleus, at ∼100 μM (44, 45, 46). Despite the mitochondrial NAD^+^/NADH ratio (∼8:1) being lower than the cytoplasmic/nuclear ratio (60–700:1) (31, 47, 48), mitochondrial PARP1 activity will have higher impact on the metabolic factor than its nuclear counterpart as a 1:1 NAD^+^/NADH was unable to inhibit PARP1 activity *in vitro* (49), suggesting NADH would, at least, need to be in excess of NAD^+^ for inhibition to be seen.

Under physiological conditions, PARP1 activity is tightly regulated. PARylation is transient in the cell, as PAR chains are rapidly degraded by PARG or other hydrolases (50). Degradation of PARP1 itself by caspases and controlled transport into mitochondria further contribute to maintaining homeostasis (18, 51). Overexpression of PARP1 in various pathological and oxidative stress conditions that cause DNA damage is well documented in literature. In such conditions, overactivated mitochondrial PARP1 depletes NAD^+^, causing metabolic disorder and cell death (15). Another outcome of overactivated mtPARP1 will be that it will inhibit mtDNA damage repair through inhibition of Pol γ, which, in turn, exacerbates OXPHOS dysfunction and reduces ATP production. Furthermore, PARP1 directly inhibits hexokinase 1 and thereby regulates glycolysis (52, 53), which can further reduce ATP level. Prolonged ATP depletion inevitably leads to necrotic cell death. Thus, mtDNA maintenance is intimately linked to cellular NAD^+^ level, metabolism, and PARP1 activity.

### PARP1 regulates Pol γ DNA repair but not DNA replication

Our studies revealed PARP1 had distinct effects on mtDNA repair and replication by Pol γ. Although conducted on a short DNA template, our results suggest that Pol γ in DNA replication is insensitive to PARP1. Contrarily, Pol γ DNA repair function is tightly regulated by PARP1 and proceeds only in the presence of sufficient NAD^+^ and competent PARylation. While both mtDNA replication and damage repair take place under physiological conditions, only replication continues when PARP1 is overexpressed, and/or metabolism is low. These conditions may occur from prolonged oxidative stress, aging processes, or degenerative diseases. Impediment of mtDNA repair and low metabolism will increase mtDNA mutations and reduce ATP production, which could lead to mitochondria-dependent apoptosis (54) and be detrimental to organ’s health.

### A model for PARP1–Pol γ interplay in mtDNA repair

Increasing evidence indicates that repair enzymes form a complex, referred to as the repairosome. The DNA repair intermediates are proposed to be handed-off among the enzymes until repair is completed. PARP1 serves as a scaffolding protein for repairosome formation. In line with this theory, we summarize our findings in the following model.

**Figure 6:**
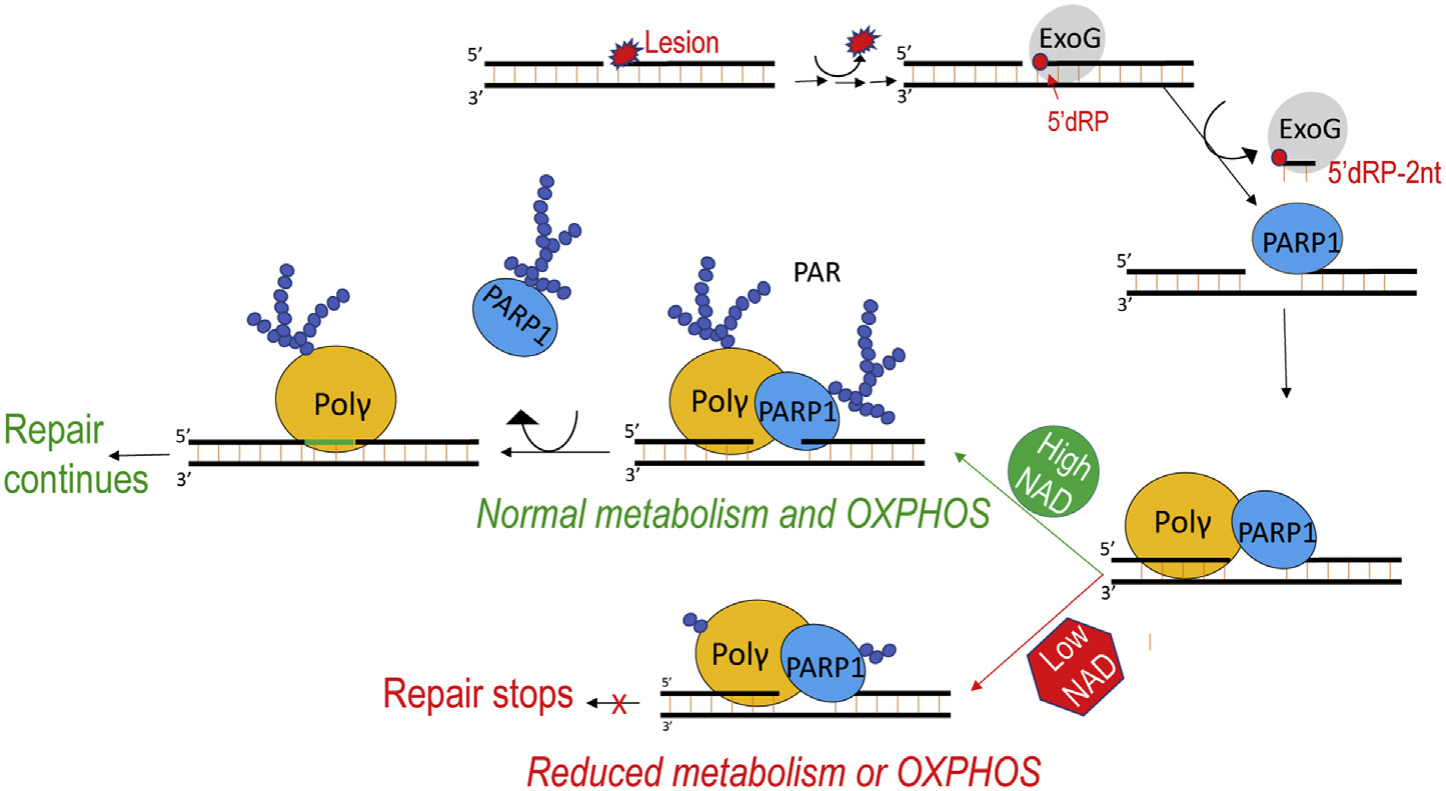
**A proposed model for coordinated activity of PARP1 and Pol γ in mitochondrial BER and the association of mtDNA repair and metabolism.** OXPHOS stands for oxidative phosphorylation electron transfer chain. BER, base excision repair; PARP1, Poly(ADP-Ribose) polymerase 1; Pol γ, mitochondrial DNA polymerase.

Upon lesion removal and DNA end cleaning, PARP1 binds the gapped DNA, followed by binding of Pol γ to the 3’-end of the gap. Because auto-PARylation drastically reduces PARP1 catalytic activity (38), in the absence of NAD^+^, PARP1 is trapped at the DNA gap, thus preventing Pol γ gap-filling synthesis. In the presence of NAD^+^, PARP1 catalyzes *trans*-PARylation of Pol γ first or simultaneously with auto-PARylation. The modified PARP1 dissociates from the DNA, thereby removing the physical hindrance to Pol γ, which is able to perform gap-filling synthesis before falling off the DNA, and BER continues (Fig. 6). *Trans*-PARylation of Pol γ will likely allow faster dissociation from DNA, granting Ligase 3 access to the gap-filled product for ligation and thereby accelerating the overall efficiency mtDNA repair.

The actual order of the events and catalytic proficiency of each respective reaction would be better illustrated by kinetics of the coupled reactions of DNA synthesis and PARylation However, the complexity of the reactions greatly exceeds the capability of steady state kinetic methods currently available, and the Michaelis–Menten kinetics conditions are unmet because (a) the product release is slow for Pol γ and PARP1 and (b) *trans*-PARylation of Pol γ depends on PARP1 and NAD^+^ concentration. Therefore, kinetic parameters of Pol γ will be functions of PARP1 and NAD^+^ instead of being numeric constants. Kinetic experiments should be carefully designed to address the coupled reaction.

Besides being a form of therapeutics to fight breast and ovarian cancer in DSBR-deficient patients, PARP1 inhibitors are also used in cardiac diseases and infection to suppress overexpressed PARP1. PARP1 overactivation plays a pivotal role in transformation of pathological cardiac hypertrophy to heart failure (55). PARP1 inhibitors improve symptoms; nevertheless, their prolonged usage could increase mutation rates (55). Our study suggests that inhibition of PARP1 catalytic activity, as well as excessive PARP1 levels, halts mtDNA repair and will exacerbate mtDNA damage. This provides a potential explanation at the molecular level for how prolonged usage of PARP1 inhibitors could stress mitochondria in cardiomyocytes and induce dysfunction. If a drug can be designed to prevent PARP1 hyperactivation or its binding to Pol γ–DNA rather than its PARylation activity, it may achieve higher efficacy with lower drug toxicity. Additionally, perhaps preventing PARP1 transport into mitochondria *via* inhibition of the Mitofilin/PARP1 interaction could overcome certain PARP1 side-effects in treatment of cardiovascular disorders. As mitochondria-specific PARP1 inhibitors have been synthesized (56), they can be used to further tease out PARP1’s function in mitochondria and nucleus.

## Experimental procedures

### Preparation of oligonucleotide substrates

Synthetic DNA oligonucleotides were purchased from Integrated DNA Technologies or Midland Certified Reagent Company. Oligonucleotide sequences are listed in Table S1.

All oligonucleotides were annealed in buffer containing 20 mM Tris (pH 8.0), 100 mM NaCl, 10% glycerol (v/v), 1 mM EDTA by heating to 95 °C followed by slow cooling to room temperature. The primer/template P25/T45 was formed with 25 nt primer annealed to the 45 nt template at 1/1.1 M ratio, and the 3 nt gap substrate T45/P25-B17 was formed by annealing T45 to P25 and a 17 nt DNA (B17) that anneals to the 5’-end of T45 at 1/1.1/1.2 M ratio. Self-complementary 75-nt dumbbell with a 3 nt gap (d3ntgap) and the 8 nt self-annealing DNA were annealed at 10 μM stock concentration (Figs. 1 and 2, Table S1).

### Protein purification

Pol γA and Pol γB purification was carried out following the previously published protocol (57). Briefly, PolγB ΔI4 variant was expressed in *E. coli* BL21-RIL and purified using Ni-NTA agarose (Qiagen) and Mono S affinity chromatography. Pol γA was expressed in Sf9 cells and purified on TALON (Clontech) and Superdex200 size exclusion columns. Purified Pol γA was mixed with Pol γB at a 1:2 M ratio and applied to the Superdex200 gel filtration column. Peak fractions corresponding to the Pol γAB holoenzyme heterotrimer were pooled and concentrated. Purity was judged to be ∼98% using SDS-PAGE. PARP-1 was expressed in *E. coli* BL21 (DE3) and purified per a published protocol (58). Following cell lysis, PARP-1 was purified by sequential application to Ni-NTA agarose (Qiagen), HiTrap Heparin HP column (GE Healthcare), and Superdex200 chromatography columns. Human Poly(ADP-ribose) glycohydrolase catalytic domain (deletion of N-terminal 455 amino acids) (hPARG-ΔN455) was purified according to Tucker *et al.* (59). After expression in *E. coli* BL21 (DE3), hPARG-ΔN455 was purified by sequential application to Ni-NTA agarose (Qiagen) and Superdex200 chromatography columns.

### Isothermal titration calorimetry

PARP-1 and Pol γ were dialyzed against Buffer RX (25 mM Hepes, pH 7.5, 140 mM KCl, 5 mM MgCl_2_, 5% Glycerol, 1 mM β-mercaptoethanol [BME]) at 4 °C overnight. Following dialysis, concentrations were determined using their respective extinction coefficients. Titrations were carried out in triplicate (PARP-1 and d3ntgap) or duplicate (PARP-1, Pol γ, and d3ntgap) using a Malvern MicroCal PEAQ-ITC at 25 °C with 19 injections (0.4 μl for the first injection, followed by 18 injections of 2 μl) of 50 μM d3ntgap titrated into 5 μM protein. For titrations involving PARP1 and Pol γ, both were 5 μM. Control titrations were performed by titrating 50 μM d3ntgap into buffer. Thermodynamic binding parameters were determined using NITPIC and SEDPHAT as described in (60). Figures for publication were produced using GUSSI (5).

### Super-shift electrophoretic mobility shift assay

Pol γ (0.5 μM) was incubated with increasing concentrations of PARP-1 (0.05 μM, 0.1 μM, 0.2 μM, 0.4 μM, 0.6 μM, 0.8 μM, 1 μM, 1.2 μM, 1.5 μM, and 2 μM) in Buffer RX on ice for 10 min. Gapped DNA substrate (d3ntgap, 0.75 μM) was added, and the protein–DNA mixtures were incubated at room temperature for 10 min. Samples were loaded onto a gradient 4 to 20% native PAGE gel (Bio-Rad), electrophoresed at 180 V for ∼1 h in 0 °C native running buffer (25 mM Tris, 192 mM glycine). Gels were stained in 1X SyBr Gold (Invitrogen) (diluted in native running buffer) and scanned using the Cy2 setting on a Typhoon Amersham Biomolecular Imager. Densitometry was performed with Image Quant TL (IQTL). The fraction of complex formed was calculated as:

$$fraction\;of\;complex=1-\frac{I_{r}}{I_{c}}$$

where *I*_*r*_ denotes the intensity of the remainder Pol γ-DNA band, and *I*_*c*_ denotes the intensity of the Pol γ–DNA band without PARP1. Because of the diffuse nature of the super-shifted species, Pol γ–PARP1–DNA complex formation is more accurately measured by the disappearance of the Pol γ–DNA binary complex. The fraction of complex formed was plotted as a function of PARP-1 concentration to obtain an apparent dissociation constant Kd.

### *Trans*-PARylation assay

Pol γ or Pol β (1 μM) was incubated with d3ntgap (0.75 μM) and increasing concentrations (2 μM, 20 μM, 200 μM, 2000 μM) of NAD^+^ in Buffer RX at 25 °C for 5 min before addition of PARP1 (1 μM) and incubated at 37 °C for 30 min. Reactions were quenched with SDS-PAGE loading buffer (60 mM Tris-HCl, pH 6.8, 2% SDS, 10% glycerol, 0.025% bromophenol blue, and 5% BME) and heated at 95 °C for 5 min. Products were resolved by electrophoresis on 10% denaturing PAGE and analyzed by WB.

Gels were incubated in transfer buffer (25 mM Tris base, 192 mM glycine, 10% methanol) at 25 °C for 10 min with gentle agitation. Reaction products were transferred to nitrocellulose membranes at 100 V for 70 min in transfer buffer; the membranes were then washed with 1X Tris-buffered saline supplemented with Tween-20 (TBST) (20 mM Tris, 150 mM NaCl, 0.05% Tween 20 [v/v]) and blocked using 1X TBST supplemented with 5% (w/v) skim milk powder for 1 h at room temperature. Membranes were then sequentially probed for 1 h with primary and secondary antibodies (details in Table S2) at room temperature. Between probes, membranes were thoroughly washed with 1X TBST. Products were visualized by chemiluminescence (Pierce ECL Western Blotting Substrate) using an ImageQuant LAS 4000.

Quantification of PARylated was calculated from the background-corrected residual of non-PARylated species (I_protein_residual_) normalized by the total intensity within the lane (I_protein_total_) using program IQTL.

$$\%\;PARylated\;protein=\left(1-\frac{I_{Protein\_residual}}{I_{Protein\_total}}\right)*100$$

### PARylation sites number determination

PAR chains on Pol γA or PARP1 were converted to mono(ADP-ribose) (MAR) by carrying out *trans*-PARylation assays similar to the above assay except that 200 μM ^32^P NAD^+^ (1.6 μCi,) was used. PARylation was stopped by addition of Olaparib (20 μM) followed by the addition of hPARG-ΔN455 (5 μM). Reaction mixtures were incubated at 37 °C for 30 min, then quenched with an equal volume of SDS-PAGE loading buffer and resolved on 10% denaturing PAGE. After autoradiography, products were visualized using an Amersham Typhoon Biomolecular imager (GE) and quantified using Image Quant TL (IQTL).

### DNA synthesis activity assays

DNA replication and gap-filling synthesis were carried out under identical conditions except the DNA substrate. A primer/template duplex (5’-^32^P-25 nt/45 nt) was used for replication and a 3 nt gapped DNA (T45/5’-^32^P25-B17) for gap-filling repair activity. 100 nM Pol γ and 200 nM PARP-1 (where indicated) were mixed with 100 nM DNA substrate in Binding Buffer (25 mM Hepes, pH 7.5, 140 mM KCl, 5% glycerol, 0.5 μg/ml BSA, 1 mM BME). Incubation was at 0 °C for 10 min and then at room temperature for 5 min. Where indicated, Olaparib (10 μM) was added during the room temperature incubation, before reaction initiation. PARylation and DNA synthesis were initiated by addition of NAD^+^, 10 mM MgCl_2_, and 50 μM dNTP. Reaction mixtures were incubated at 37 °C for 5 min, quenched with stop buffer (80% formamide, 50 mM EDTA, 0.1% SDS, 5% glycerol, 0.02% bromophenol blue), heated at 95 °C for 5 min, separated on 20% PAGE/7 M Urea gels in 1X TBE at 15 W for ∼1.5 h, and autoradiographed. The substrate and product were visualized on an Amersham Typhoon Biomolecular imager (GE) and quantified using ImageQuant. Fraction of product was calculated using the intensities of DNA substrate (I_substrate_) and product (I_product_)

$$fraction\;of\;product=\frac{I_{product}}{I_{product}+I_{substrate}}$$

### Purification of PARylated Pol γ

*Trans*-PARylation of Pol γ (1 μM) was catalyzed by PARP-1 (3 μM) and 200 μM NAD^+^ with d3ntgap DNA substrate (0.75 μM) at 37 °C for 30 min. KCl concentration in the reaction mixture was increased to 500 mM to dissociate protein–DNA complexes. The reaction mixture was applied to a TALON column, which was subsequently washed with wash buffer (20 mM Hepes, pH 7.5, 200 mM KCl, 5% glycerol, 5 mM imidazole) to remove the d3ntgap DNA and free NAD^+^. The His-tagged modified and nonmodified Pol γ and PARP1 were eluted with elution buffer (20 mM Hepes, pH 7.5, 100 mM KCl, 5% glycerol, 200 mM imidazole) and was applied to a Mono S cation exchanger chromatography column (low salt buffer: 20 mM Hepes, pH 7.5, 60 mM KCl, 5% glycerol, 5 mM BME; high salt buffer: 20 mM Hepes, pH 7.5, 700 mM KCl, 5% glycerol, 5 mM BME), where the negatively charged PAR-proteins were separated in the flow-through from the bound native enzymes. The flow-through was collected and concentrated by centrifugation in a Vivaspin6 (MWCO: 100,000) at 1000 RPM for 10 min at 4 °C. At each step of the purification procedure, samples were tested by WB to verify separation of PARylated from non-PARylated Pol γ. Concentration of modified Pol γ was determined by treating an aliquot of PARylated enzyme with hPARG, then running the MARylated sample on a calibration gel containing samples of known concentrations. Densities for the bands of known concentrations were measured and graphed, allowing calculation of the concentration of the MARylated band.

### Liquid chromatography tandem mass spectrometry

The *trans*-PARylated proteins were denatured in 1% SDS at room temperature. To each 22 μl denatured sample, an equal volume of 10% SDS in 100 mM TEAB buffer (pH 8.0) was added, followed by 1 μl of 0.25 M Bond-Breaker tris(2-carboxyethyl)phosphine solution (ThermoFisher). The mixture was incubated at 56 °C for 30 min. After samples cooled to room temperature, 0.5 M Iodoacetamide (BioUltra) was added and incubated at room temperature for 30 min in the dark. 2.7 μl of 12% phosphoric acid was added in the dark, followed by 165 μl of 90% methanol in 100 mM TEAB buffer. The solution was applied to a micro S-Trap column and centrifuged for 1 min at 1200 cpm. The S-Trap was then washed sequentially with 100 mM TEAB buffer, 90% methanol in 100 mM Hepes (pH 8.5), and 150 mM NaCl in 100 mM Hepes (pH 8.5). 0.5 M NH_2_OH was applied to the S-Trap and incubated at room temperature overnight. The S-trap was washed twice with TEAB buffer before adding 25 μl of 20 ng/μl Trypsin (in 50 mM TEAB) and incubating at 47 °C for 2 h. Tryptic peptides were eluted from the S-Trap with 40 μl of 50 mM TEAB, 40 μl of 0.2% formic acid (FA), 30 μl of 50% acetonitrile (ACN) with 0.2% FA, and 30 μl of 80% ACN with 0.1% FA. The eluted tryptic peptides were dried in a speed vac and reconstituted with 100 μl of 2% ACN and 0.1% FA for mass spectrometry.

Nano-LC/MS/MS was performed on a Thermo Scientific Orbitrap Fusion system coupled with a Dionex Ultimat 3000 nano HPLC and auto sampler with 40 well standard trays. The sample was injected onto a trap column (300 μm i.d. × 5 mm, C18 Pep Map 100, Thermo) followed by a C18 reverse-phase nano LC column (Acclaim PepMap 100 75 μm × 25 cm, Thermo), both of which had previously been heated to 50 °C in the chamber. The flow rate was set to 400 nl/min with a 60-min gradient, where mobile phases were A (99.9% water, 0.1% FA) and B (99.9% ACN, 0.1% FA). As peptides eluted from the column, they were sprayed through a charged metal emitter tip into the mass spectrometer. Parameters included the following: tip voltage +2.2 kV, fourier transform mass spectrometry mode was set for MS acquisition of precursor ions (resolution 120,000); ion trap mass spectrometry mode was set for subsequent MS/MS *via* higher energy collisional dissociation on top speed every 3 s.

Proteome Discoverer 2.4 was used for protein identification and peak area detection. The UniProt human database (75,777 entries as of December 2020 when experiments were performed) was used to analyze raw data. The digestion enzyme was set to trypsin, and carbamidomethyl cysteine was as a fixed modification, and oxidized methionine and PARylated aspartate and glutamate were set as dynamic modifications. A maximum of two missed cleavages was allowed. The precursor mass tolerance was set to 10 ppm, the MS/MS fragment mass tolerance was 0.2 Da, and peptides with +2, +3, and +4 charges were considered. The resulting peptides are considered as significant when the false discovery rate was determined using Percolator and is ≤1%.

### Quantification and statistics

All assays were performed three or more times independently, the results from each experiment were used to calculate the mean and standard deviation $\left(\text{std}=\sqrt{\frac{\sum {\left(x-\bar{x}\right)}^{2}}{\left(n-1\right)}}\right)$, the maPlot.

## Data availability

All mass spectrometry data described in this manuscript (raw and mzTab spectra) are located in the MassIVE database (depository identifier MSV000086606).

## Conflicts of interest

The authors declare no conflicts of interest in regards to this manuscript.
