# Supplementary material for: Poly(ADP-ribose) polymerase 1 regulates mitochondrial DNA repair in an NAD-dependent manner
Source: J Biol Chem. 2021 Jan 19;296:100309. doi: 10.1016/j.jbc.2021.100309 (PMC7949115; doi:10.1016/j.jbc.2021.100309)

SUPPORTING INFORMATION

Table S1. Oligonucleotide sequences

Name	Sequence
75mer dumbbell 3nt gap (d3ntgap)	5'-CTTTTCTGGTGAAAAGCTCGTCGGCAGCGCTTGAGCAGCGGCAGCTGGTGCTGCCGC TGCTCAAGCGCTGCCGAC-3'
8nt self-annealing oligo	5'-GGAATTCC-3'
25mer primer (P25)	5'-CGAAAACGAGGGCCAGTGCCATACC-3'
45mer template (T45)	5'-TACGAGCCTGCCTGACGTGCGGTATGGCACTGGCCCTCGTTTTCG-3'
17mer blocker (B17)	5'-CGTCAGGCAGGCTCGTA-3'

Table S2. Antibodies, sources, and titers.

Primary Antibody			Secondary Antibody		
Antibodies	Manufacture	Dilution	Host, against	Manufacture	Dilution
Mouse, anti-PARP1	ThermoFisher	1:2,000	Goat, anti-mouse	Abcam	1:5,000
Rabbit, anti-Polγ A	ThermoFisher	1:2,000	Goat, anti-rabbit	ThermoFisher	1:5,000
Mouse, anti-PAR	Enzo	1:2,000	Goat, anti-mouse	Abcam	1:5,000
Rabbit, anti-Polβ	Abcam	1:2,000	Goat, anti-rabbit	ThermoFisher	1:5,000

Table S3. PARylation sites detected by mass spectrometry

Without NAD^+:

Protein	Peptide Sequence	Modification	Amino Acid #	m/z [Da] (by Search Engine): MS Amanda 2.0	m/z [Da] (by Search Engine): Sequest HT	Precursor Charge	XCorr (by Search Engine): Sequest HT	Amanda Score (by Search Engine): MS Amanda 2.0
PARP-1	KMVdPEKPQLGMIDR	D4(Parylation)	145	443.735	443.735	4	3.9	132.02
	NTHATTHNAYDLeVIDIFKIER	E13(Parylation)	832		523.866	5	4.95	
PolγA	LSDEGeWLVR	E6(Parylation)	183	406.875	406.875	3	3.14	108.33
	YGPEGEAVPVAIPEeR	E15(Parylation)	192		864.439	2	3.87	
	dWQEQLVVGHNVSFDR	D1(Parylation)	260	648.650	648.650	3	5.36	210.52
	RGPAISSWdWLDISSVNSLAEVHR	D9(Parylation)	346		904.120	3	5.53	

	MedGTLQAGPGGASGPR	E2(Parylation); D3(Parylation)	775 & 776		815.879	2	3.69	
PolγB	SHAELEGNGEHPEAPGSGEGSEALLeIcQR	E26(Parylation); C28(Carbamidomethyl)	71		794.611	4	4.58	

With NAD$^+$:

Protein	Peptide Sequence	Modification	Amino Acid #	m/z [Da] (by Search Engine): MS Amanda 2.0	m/z [Da] (by Search Engine): Sequest HT	Precursor Charge	XCorr (by Search Engine): Sequest HT	Amanda Score (by Search Engine): MS Amanda 2.0
PARP-1	KMVdPEKPQLGMIDR	D4(Parylation)	145	443.735	443.735	4	4.41	169.06
	eELGFRPEYSASQLK	E1(Parylation)	168	590.300	590.300	3	3.97	124.89
	GFSLLATeDKEALKK	E8(Parylation)	190	555.645	555.645	3	3.49	187.38
	KGDeVDGVDEVAK	E4(Parylation)	212	688.338	459.228	3	3.9	223.78
	VADGMVFGALLPceEcSGQLVFK	C13(Carbamidomethyl); E14(Parylation); C16(Carbamidomethyl)	296		848.076	3	5.49	
	SLQeLFLAHILSPWGAEVKAEPVEVVAPR	E4(Parylation)	471		800.941	4	8.11	
	SLQELFLAHILSPWGAeVK	E17(Parylation)	484	1077.089	1077.091	2	6.14	379.3
	AEPVeVVAPR	E5(Parylation)	491	541.304	541.304	2	2.37	135.92
	GGAAVdPDSGLEHSAHVLEK	D6(Parylation)	534	501.751	501.751	4	3.76	112.27
	LQLLEDDKeNR	E9(Parylation)	580	463.244	463.244	3	3.37	191.12
	FYPLeIDYGQDEEAVK	E5(Parylation)	642	965.957	965.957	2	2.89	149.98
	KFYPLeIDYGQDEEAVKK	E6(Parylation)	642	729.704	729.704	3	5.79	245.92
	KFYPLeIDYGQDEEAVK	E6(Parylation)	642	687.006	687.006	3	3.8	150.7
	QIQAAYSILSeVQQAVSQGSSDSQILDLSNR	E11(Parylation)	715		1117.567	3	2.91	
PolγA	GLHEQIFGQGGeMPGEAAVRR	E12(Parylation)	93		564.284	4	3.55	
	GLHEQIFGQGGEMPGeAAVR	E16(Parylation)	97	700.010	700.010	3	5.16	263.6
	YGPeGEAVPVAIPEER	E4(Parylation)	181	864.433	864.433	2	3.83	242.74
	dWQEQLVVGHNVSFDR	D1(Parylation)	260	648.650	648.650	3	4.42	200.3
	eNFQDLmQYcAQdVWATHeVFQQQLPLFLER	E1(Parylation); M7(Oxidation); C10(Carbamidomethyl);	387, 399, 405		1315.619	3	2.18	

		D13(Parylation);<br>E19(Parylation)						
	SLMDLANdAcQLLSGER	D8(Parylation);<br>C10(Carbamidomethyl)	469	509.753		4		56.66
	LPIeGAGAPGDPMDQEDLGPcSEEEEFQQDVMAR	E4(Parylation);<br>C21(Carbamidomethyl)	516		1250.205	3	6.37	
	LPIEGAGAPGDPMDQEdLGPcSEEEEFQQDVmAR	D17(Parylation);<br>C21(Carbamidomethyl);<br>M32(Oxidation)	529		1250.205	3	5.17	
	LMALTWdGFPLHYSER	D7(Parylation)	608	488.494	488.494	4	2.67	130.38
	DNLAKLPTGTTLeSAGVVcPYR	E13(Parylation);<br>C19(Carbamidomethyl)	641		793.077	3	5.18	
	LPTGTTLeSAGVVcPYR	E8(Parylation);<br>C14(Carbamidomethyl)	641	918.470	918.470	2	4	246.55
	dFLPKMEDGTLQAGPGGASGPR	D1(Parylation)	769		739.364	3	3.51	
	LSDeGEWLVR	E4(Parylation)	1000	609.809	609.809	2	3.21	121.11
PolyB	SHAeLeGNGEHPEAPGSGEGSEALLEIcQR	E4(Parylation);<br>E6(Parylation);<br>C28(Carbamidomethyl)	49 &<br>51		798.363	4	4.67	
	SHAELeGNGEHPEAPGSGEGSEALLEIcQR	E6(Parylation);<br>C28(Carbamidomethyl)	51		794.612	4	6.02	
	FAMSPSNFSSSdcQDEEGR	D12(Parylation);<br>C13(Carbamidomethyl)	277	1083.428	1083.428	2	4.39	316.62
	KFAMSPSNFSSSdcQDEEGR	D13(Parylation);<br>C14(Carbamidomethyl)	277	765.320	765.320	3	6.89	222.75
	GmLAYLYdSFQLTENSFTR	M2(Oxidation);<br>D8(Parylation)	352	1144.039	1144.039	2	2.11	65.16
	GMLAYLYDSFQLTeNSFTR	E14(Parylation)	358	1136.041	1136.041	2	3.77	310.56

SUPPORTING FIGURES

Figure S1 Pol γ-PARP1 interaction determined by ITC. Measurements were carried out on gap-DNA binary complexes with PARP1 (A), Pol γ (B) and ternary complex with PARP1 and Pol γ (C). Fitting residals for each complex is displayed at lower panel. Each experiment was performed in duplicate or triplicate. D) Superposition of analyzed data for PARP1-DNA and PARP1-Pol γ-DNA complexes. E) Titration of PARP1 into Pol γ in the absence of DNA.

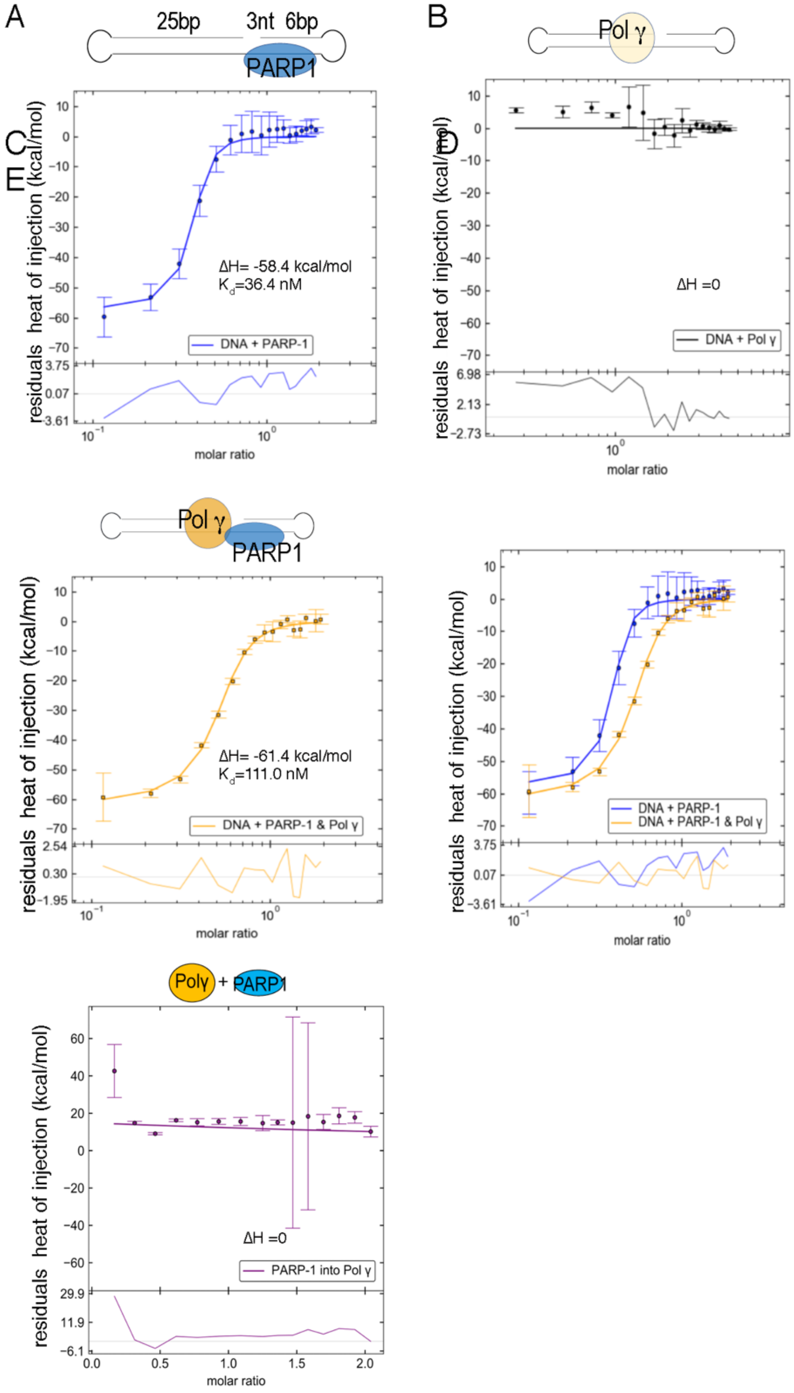

Figure S2 EMSA supershift of Pol γ-DNA-PARP1 complex formation A) Titrating toPol γ-DNA complex with PARP1 at 50, 100, 200, 400, 600, 800, 1000, 1200, 1500, and 2000 nM concentrations. B) quantification of A), where the estimated K_d for PARP1-Pol γ-DNA complex is ~600 nM. C) Western Blot of EMSA with antibody against Pol γ. All samples contain 0.75 µM 3nt- gapped DNA (d3ntgap) without (Lane 1-5) or with Olaparib (Lane 6-10), 0.5 µM Pol γ, 1 µM PARP1 and 2 mM NAD^+. Lane M is a size marker.

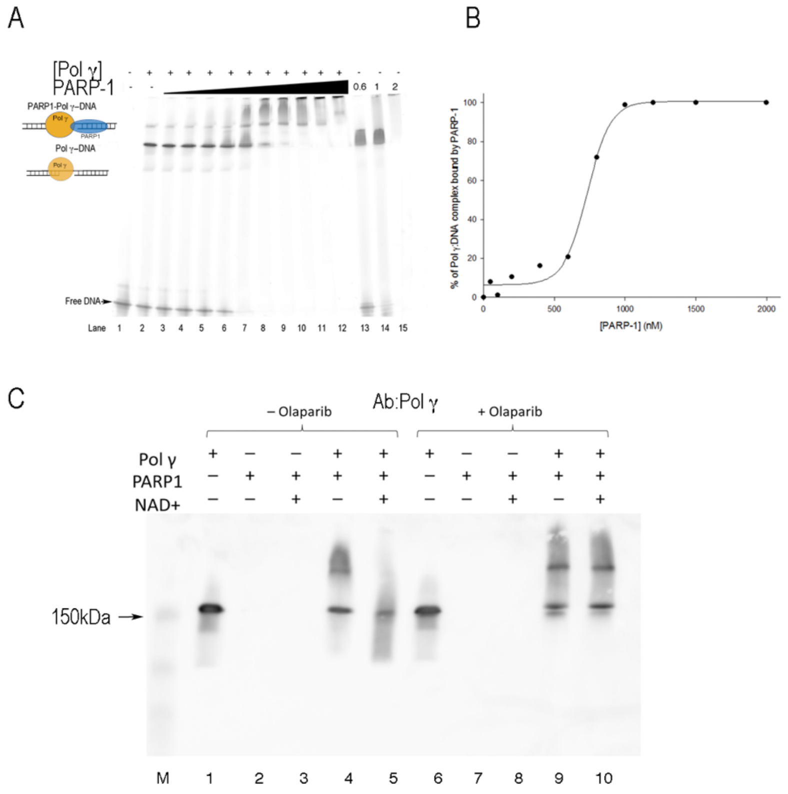

Figure S3. Effect of PARP on Pol β gap-filling activity. A) Pol β activity was analyzed on the 3-nt gapped DNA T45/P25-B17 (Table S1) in the absence of PARP1 (Lane 1), and in the presence of PARP without NAD^+ (Lane 2) and with increasing concentrations (2, 20, 200 and 2000 μM) of NAD (Lanes 3-7), and in the presence of PARP1 inhibitor, Olaparib (Lane 8). Lane M and S are marker and substrate only, respectively. B) quantification of Pol β product as a function of NAD (plotted the mean of four experiments with standard deviation) C. Western Blot analysis carried out with antibody against human Pol b in reactions containing Pol b and the gapped DNA without NAD (Lane 1), and with PARP1 without NAD+ (Lane 2) and with 2, 20, 200 and 2000 μM NAD (Lanes 3-6).

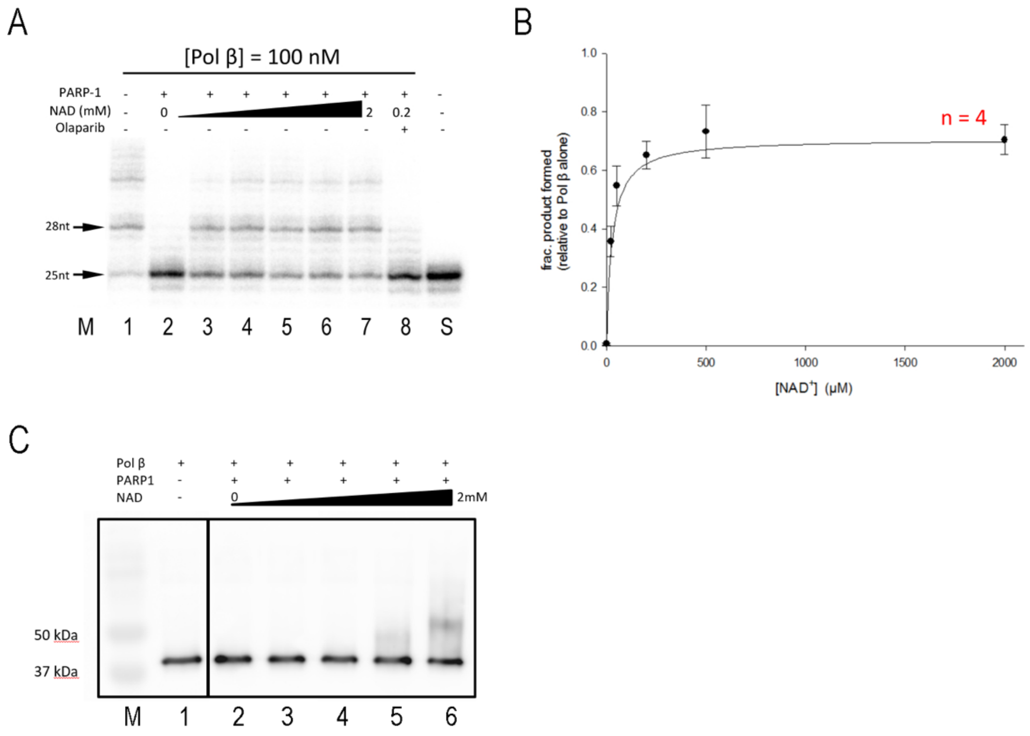

Figure S4. Determination of auto- and trans-PARylation sites numbers. Samples contained 1 µM PARP1, 1 µM Pol γ, 0.75 µM dumbbell 3-nt gapped DNA, and 200 µM NAD^+ (spiked with ^{32}P NAD^+). The PARylation reaction was quenched by addition of Olaparib, PAR chains were digested to mono(ADP-Ribose) using hPARGΔ455, and samples were run on SDS-PAGE. Mono (^{32}P-ADP-ribose) (MAR) modified Pol γA, Pol γB and PARP1 were quantified and normalized to the density of the MAR-PARP1 band in lane 3 (PARP1 alone).

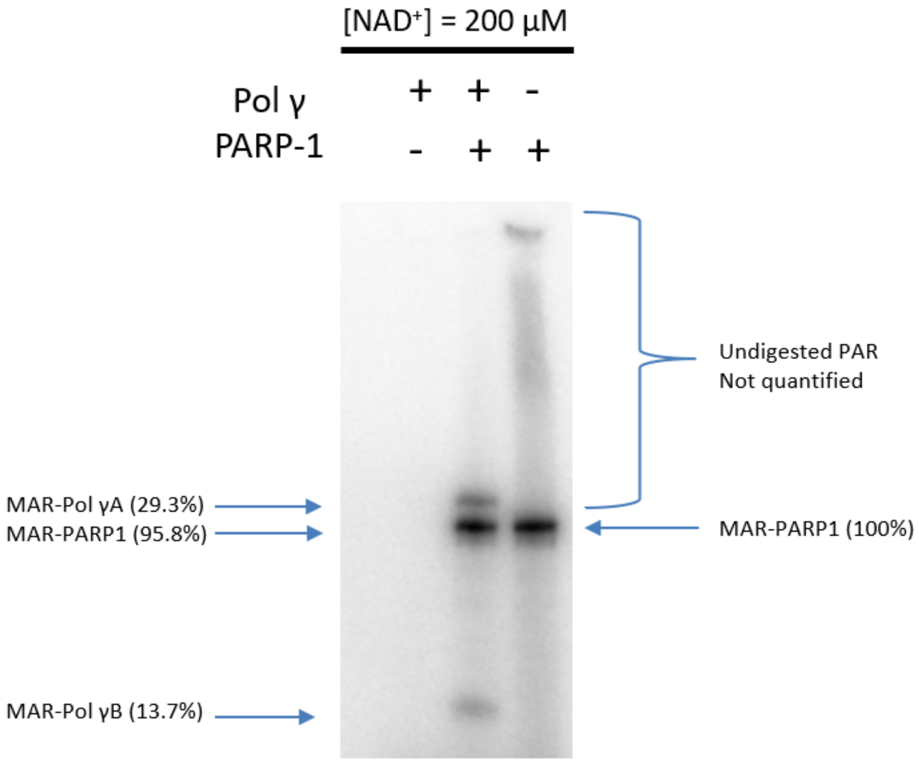

Figure S5 Separation and Gap-filling activity of PARylated Pol γ   A) Chromotograph of resolving PARylation reaction mixture that contains modified (PAR-) and unmodified Pol γ, PARylated and unmodified PARP1. B) Fractions of the A) analyzed on a denaturaing PAGE. Lane 1, PARylation reaction mixture, Lane 2-12 are fractions of Peak 1, Lane 12 was taken from Peak 2. The smeares are characteristics of a PARylated protein. C) gap-filling reaction products from unmodified and modified Pol γ. D) quantification of C) where fraction of product is determined (1- background corrected substrate fraction Fs), where Fs=Counts of substrate DNA.

An added note that unmodified Pol γ is consistently eluted at the location of Peak 2, offering support that unmodified Pol γ was separate from the modified species. Our method did not remove PARylated PARP1 during the purification process. As PARylated PARP1 has negletable affinity to DNA, it should have not effect on gap-filling synthesis.

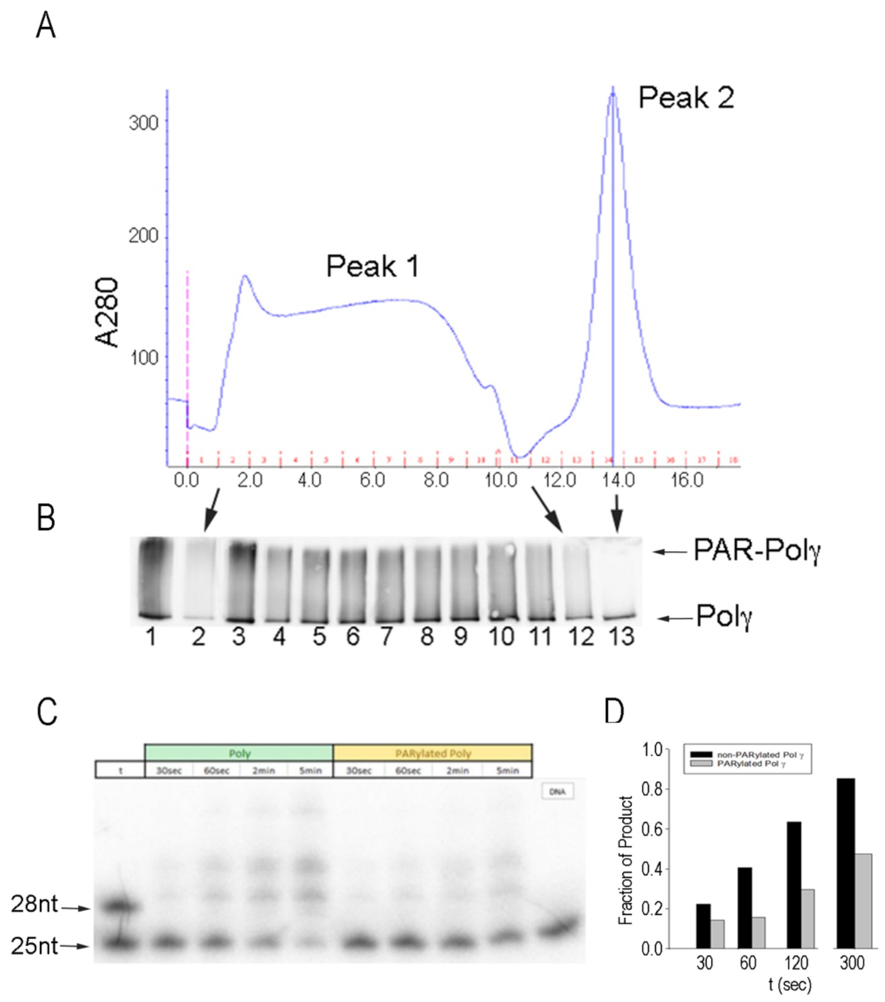

Figure S6 DNA depedent parylation Trans-PARylation assays were performed by incubating Pol γ (1 μM) the indicated gapped DNA substrate (0.75 μM or no DNA) and NAD concentration prior to addition of PARP1 (1 μM).

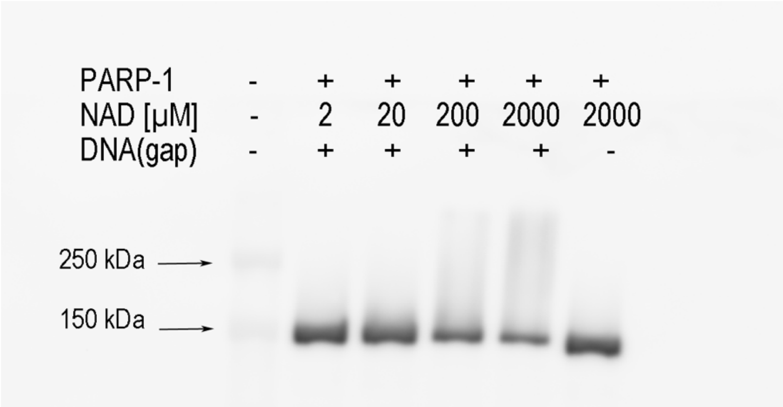

Supplement: Supplemental Figures S1–S5 and Tables S1–S3 [file mmc1.pdf]
